# Reference Gene and Protein Expression Levels in Two Different NAFLD Mouse Models

**DOI:** 10.1155/2020/1093235

**Published:** 2020-02-03

**Authors:** Layanne C. C. Araujo, Silvana Bordin, Carla R. O. Carvalho

**Affiliations:** Department of Physiology and Biophysics, Institute of Biological Science, University of Sao Paulo, Sao Paulo 05508-900, Brazil

## Abstract

The expression levels of some reference genes and proteins are used for data normalization and quantification. However, these levels can change in response to experimental conditions or treatments. *Aim*. The aim of this work was to evaluate reference gene and protein expression in models of nonalcoholic fatty liver disease, using mice fed with a high-fat diet (HFD) and mice that are genetically obese (ob/ob). *Main Methods*. Histological staining techniques were used to verify the morphology and quantify the amount of lipid droplets present in the liver. Real-time polymerase chain reaction and immunoblotting were employed for monitoring protein expression and gene expression levels, respectively. *Key Finding*. The results showed that there was a substantial increase in the amount of lipid droplets in the livers of HFD and ob/ob animals when compared to the standard diet (SD) group. There was an observed reduction in the expression of *β*-actin (10%), *α*-tubulin (6%), GAPDH (19%), and RPL3 (15%) genes when comparing the ob/ob group to the HFD group. Additionally, the ob/ob mice displayed GAPDH protein levels that were substantially, but not significantly, reduced when compared to SD. *Significance*. It was concluded that there are slight differences in the expression levels of reference genes and proteins in these two NAFLD animal models, and researchers should consider these alterations when working with these models.

## 1. Introduction

Qualitative and quantitative analyses, of gene and protein expression, rely on the presence of reference genes and proteins that are expressed at constant levels in the analyzed samples. Inappropriate endogenous controls can compromise the accuracy and reliability of the results. This is because these genes and proteins are commonly used to normalize data and correct experimental errors, thus allowing for direct comparisons of gene and/or protein expression [1].

Previous studies have shown that the expression of some internal controls varies, depending on the experimental conditions. For example, the expression levels of *β*-actin, glyceraldehyde-3-phosphate dehydrogenase (GAPDH), and *β*-tubulin were shown to increase in some tumors [1, 2], while other conditions such as Alzheimer's disease, alcoholic hepatitis, cirrhosis, and schizophrenia have been implicated in altering the expression of endogenous reference genes and/or proteins [3, 4]. Furthermore, nonalcoholic fatty liver disease (NAFLD) has also been shown to alter the constitutive expression of reference genes used to normalize gene expression levels [5].

In a study previously performed by our research group with high-fat diet and ob/ob mice, which we used as NAFLD models, it was used as constitutive the *β*-actin for protein expression and *β*2 microglobulin (B2M) for gene expression [6]. As it was seen that these constitutive genes and proteins were suitable for experiments with these models and that there are no standardization studies for constitutive use in NAFLD, we set out to investigate different genes and proteins.

Studies based on the NAFLD model use different constitutive genes and proteins for gene and protein expression, and some use *β*-actin and others GAPDH and *α*-tubulin; there is no standard constitutive genes and proteins for the model [7–9].

Based on that, the goal of the present study was to analyze the gene and protein expression levels of some commonly employed endogenous controls in hepatic steatosis model triggered by a high-fat diet (HFD) or genetic obesity (ob/ob mice). The results will be discussed in terms of the utility of each control, and recommendations about reference gene and protein selection when working with these models are provided.

## 2. Material and Methods

We followed the methods of Layanne C. da C. Araujo et al. [6].

### 2.1. Animals

Male C57BL/6 mice, weighing 20-25 g, were obtained from the vivarium at the University of São Paulo (USP) Medical School and maintained in a temperature controlled room at 22 ± 2°C. The mice had free access to food and water and were subjected to a 12-hour light-dark cycle (lights on from 6 am to 6 pm). The genetically obese ob/ob mice, weighing 55-60 g, were obtained from the Department of Physiology and Biophysics at the Institute of Biomedical Sciences of USP (ICB-USP) and kept under the same conditions as described above. Each group consisted of 10 animals and the statistical analysis performed considered at least 5 animals per group. Standard diet (SD) consisted of commercial rodent chow (Nuvilab® CR-1, Curitiba, Parana, Brazil), which contained 22% protein, 61% carbohydrate, 4.5% fat, 8% cellulose, and 4.5% vitamins and minerals, providing 3.2 kcal/kg chow. The HFD chow (PragSoluçoes Biosciences, Jau, Sao Paulo, Brazil) consisted of pellets containing 20.3% protein (15% kcal/kg), 36% carbohydrate (27% kcal/kg), 34% fat (57% kcal/kg), 4.7% vitamins and minerals (0.7% kcal/kg), and 5% fibers. The animals were fed with HFD for 8 weeks. All experimental procedures were performed following the principles of the “Guidelines for the ethical use of animals in applied etiology studies” [10] and this study was previously approved by the Ethics Committee on the Use of Animals at ICB-USP (Protocol No. 035, in page 30, book 03).

## 3. Liver Morphology

### 3.1. Hematoxylin and Eosin Staining

To evaluate liver morphology, samples were placed in individual cassettes, fixed in a 10% formaldehyde solution for 8 hours, transferred to 70% alcohol, and stored overnight. The samples were then dehydrated through a series of baths in 95% alcohol and 100% alcohol and xylene. Following dehydration, the tissue samples were embedded in paraffin at 60°C. A microtome (Zeiss, Jena, Germany) was used to cut the samples into five micron slices, which were then stained with hematoxylin and eosin (H&E). Nikon Eclipse Ti-U microscope was used with Nikon DS-Ri1 digital camera and NIS-Elements BR 3.1 software.

### 3.2. Oil Red O Staining

Alternatively, the paraffin-embedded liver samples were placed in isopropanol and immediately frozen in liquid nitrogen. Twelve micron slices were prepared using a cryostat (Microm H560 Thermo Scientific, Massachusetts, USA). Three slices from different parts of the sample were placed on each slide, and two slides per animal were used. The slides were stained with Oil Red O (ORO) and Mayer's hematoxylin. Ten images from each animal were obtained at 20x magnification. The identification of the ORO-stained area was performed with the ImageJ program [11].

### 3.3. Real-Time Polymerase Chain Reaction

Total RNA from the liver was extracted with TRIzol reagent (Thermo Scientific, Massachusetts, USA) and reverse transcribed to cDNA (High-Capacity cDNA Kit, Applied Biosystems, USA). Gene expression was evaluated by real-time polymerase chain reaction (RT-PCR), using Rotor Gene Q (Qiagen, USA) and the fluorescent dye SYBR Green (Platinum® SYBR® Green qPCR Supermix UDG, Invitrogen, USA). The primer sequences were *β*-actin forward: 5′-TCAAGATCATTGCTCCTCCTG-3′, reverse: 5′-GCTCAGTAACAGTCCGCCTAG-3′; *α*-tubulin forward: 5′-TATGCCAAGCGTGCCTTTG-3′, reverse 5′-CACAGAATCCACACCAACCTCC-3′; GAPDH forward: 5′-AAGGTGGTGAAGCAGGCATC-3′, reverse: 5′-CGAAGGTGGAAGAGTGGGAG-3′; RPL37a forward: 5′-GTACACTTGCTCCTTCTGTGGC-3′, reverse: 5′-AGGTGGTGTTGTAGGTCCAGG-3′; *α*-actinin forward: 5′-CCGCACCATCAATGAGGTCG-3′, reverse: 5′-AGGCTTGGAAGGTCACAACCC-3′; B2M forward: 5′-CCCCACTGAGACTGATACATACG-3′, reverse: 5′-CGATCCCAGTAGACGGTCTTG-3′. The analysis of gene expression was carried out using a method previously described by Livak and Schmittgen [12] and Pfaffl [13].

### 3.4. Western Blot Analysis

Animals were deeply anaesthetized with thiopental (50 mg/kg b.w.); after loss of corneal reflexes, the abdominal wall was opened to visualize the liver and portal vein. The animals were then euthanized, and the livers were removed and homogenized in lysis buffer (100 mM Tris, 10% SDS, 100 mM sodium pyrophosphate, 100 mM sodium fluoride, 10 mM EDTA, 10 mM sodium orthovanadate). The tissue extracts were centrifuged at 15,294 × *g*, at 4°C, for 40 minutes. The protein content of the supernatants was measured using the Bradford method. For SDS-PAGE, 50 *μ*g of total protein was mixed with Laemmli buffer containing 200 mM dithiothreitol, loaded onto a 10% gel, and separated at 120 V for 120 minutes. The resolved gels were then transferred to nitrocellulose membranes using a Bio-Rad mini trans blot apparatus (USA). The membranes were incubated in blocking solution (5% skim milk) for 2 hours at room temperature. The membranes were then incubated, with primary antibodies, overnight at 4°C, and washed and incubated with a horseradish peroxidase-conjugated secondary antibody. The specific antibodies used included anti-: *β*-actin (catalog number 4970S) and *α*-actinin (catalog number 3134S) (Cell Signaling, Massachusetts, USA), *α*-tubulin (catalog number 322500) (Thermo Scientific, Massachusetts, USA), and *α*-GAPDH (catalog number sc-25778) (Santa Cruz, Dallas, USA). Ponceau S staining was used to calculate the relative protein content. The visualization of the immunoblots was accomplished with enhanced chemiluminescence (Amersham Biosciences). The immunoblots were scanned and quantified using the ImageJ software.

## 4. Statistical Analysis

The results were analyzed using GraphPad Prism v7.0 (GraphPad Software, La Jolla, CA, USA). The minimum sample size per group for each parameter analyzed was defined by an *n* sufficient to perform the analysis of distribution of samples through the “D'Agostino and Pearson omnibus normality test,” as recommended by the GraphPad software. All samples were evaluated for normal distribution and subjected to a one-way ANOVA followed by the post-hoc Bonferroni test (Bonferroni multiple comparison test) (*p* < 0.05). The results were expressed as the mean ± standard error of mean (mean ± SEM).

## 5. Results

### 5.1. Morphology

**Figure 1:**
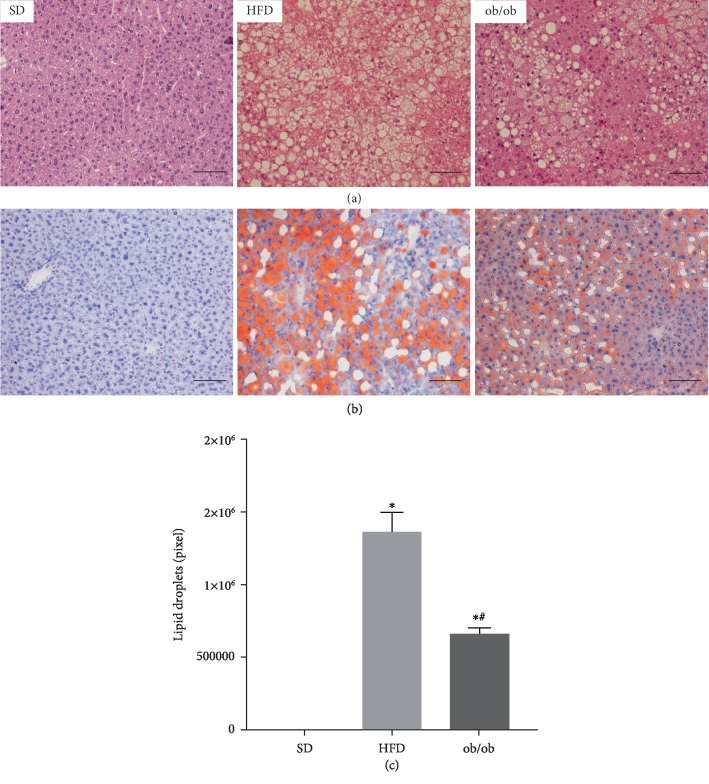
Lipid droplet content in the livers of standard diet (SD), high-fat diet (HFD), and genetically obese (ob/ob) mice. (a) H&E-stained samples and (b) ORO-stained samples. Representative images of the livers of animals fed with a standard, high-fat diet and ob/ob ((a) H&E and (b) ORO, 20x magnification). The results are represented as the mean ± SEM. The statistical differences as indicated by one-way ANOVA were as follows: ^∗^*p* < 0.05 (SD *vs*. HFD; SD *vs*. ob/ob), ^#^*p* < 0.05 (HFD *vs*. ob/ob), *n* = 5.

As shown in Figure 1, there were no detectable lipid droplets detected in liver samples of the SD mice, as evidenced by H&E staining (top left panel). In contrast, the hepatocytes of HFD (top middle panel) and ob/ob (top right panel) mice contained substantial amounts of intracellular lipid droplets. For a more quantitative assessment of the lipid content, the color intensity of the ORO stained samples was measured (1.4 × 10^6^ ± 1.0 × 10^5^ and 6.7 × 10^5^ ± 3.4 × 10^4^ pixels, respectively).

### 5.2. Gene Expression

**Figure 2:**
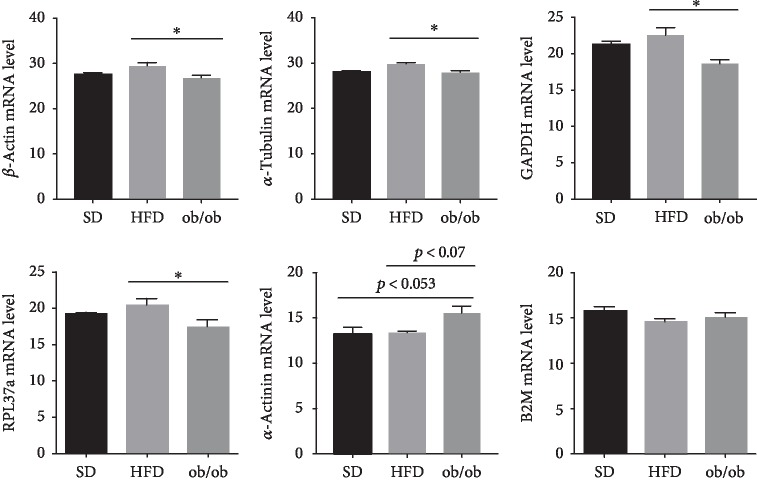
Levels of constitutive gene expression in the livers of standard diet (SD), high-fat diet (HFD), and genetically obese (ob/ob) mice. The results are represented as the mean ± SEM. The statistical differences as indicated by one-way ANOVA were as follows: ^∗^*p* < 0.05 (HFD *vs.* ob/ob), *n* = 6‐10.

The results of the RT-PCR analyses (Figure 2) failed to detect any statistically significant differences in the levels of constitutive gene expression of *β*-actin, *α*-tubulin, GAPDH, RPL3, and B2M between or among groups. However, when comparing the ob/ob group to the HFD group, there was an observable reduction in the amounts of *β*-actin (10%), *α*-tubulin (6%), GAPDH (19%), and RPL3 (15%). On the other hand, ob/ob mice also presented a substantially elevated levels of *α*-actinin expression when compared to the SD and HFD groups, but this result failed to reach a level of statistical significance (*p* = 0.053).

### 5.3. Protein Expression

**Figure 3:**
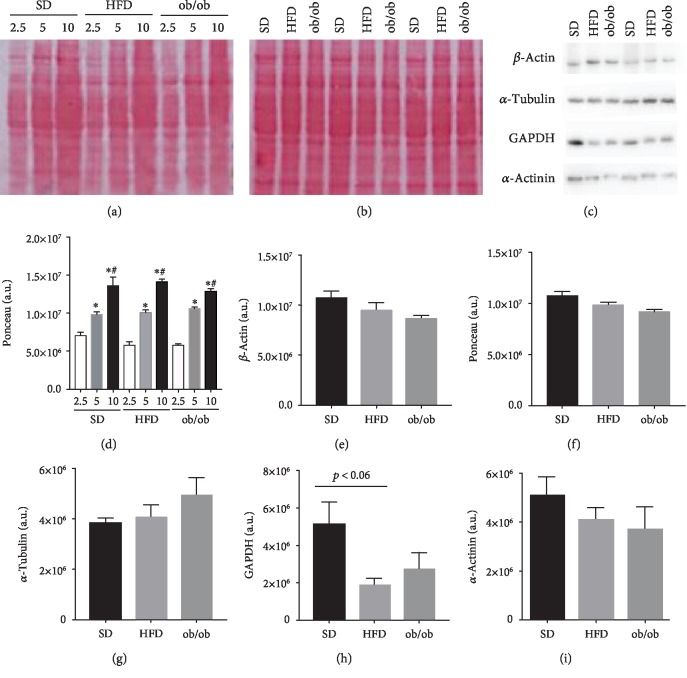
Levels of constitutive protein expression in the livers of standard diet (SD), high-fat diet (HFD), and genetically obese (ob/ob) mice. (a) Ponceau S: different protein concentrations (2.5, 5, and 10 *μ*g/*μ*L). (b) Ponceau S: protein concentration of 5 *μ*g/*μ*L. Representative image of proteins (c). The results are represented as the mean ± SEM. (d–i) The statistical differences as indicated by one-way ANOVA were as follows: ^∗^*p* < 0.05 (SD2.5 *vs*. SD5; SD2.5 *vs*. SD10; HFD2.5 *vs*. HFD5; HFD2.5 *vs*. HFD10; ob/ob2.5 *vs*. ob/ob5; ob/ob2.5 *vs*. ob/ob10), ^#^*p* < 0.05 (SD5 *vs*. SD10; HFD5 *vs*. HFD10; ob/ob5 *vs*. ob/ob10), *n* = 6‐10.

Figures 3(a) and 3(d) demonstrate that the intensity of Ponceau S staining increases as more protein is loaded onto the gel and subsequently transferred. Note the increased intensity in staining when larger amounts of protein were loaded onto the gel (2.5 vs. 5 vs. 10 *μ*g/*μ*L). However, with similar protein loads, from each group of mice, there were no statistically significant differences between or among groups (Figures 3(b) and 3(e)).  Figure 3(c) shows a typical immunoblot using each specific antibody.

With regard to protein expression, there were no significant differences detected in the levels of *β*-actin, *α*-tubulin, *α*-actinin, or GAPDH between groups (Figures 3(f)–3(h)). However, the GAPDH protein levels in the livers of HFD mice were considerably reduced (64% ± 19; *p* < 0.06) and the levels in the ob/ob mice were reduced by approximately half (53 ± 33%; not significant), when compared to SD mice.

## 6. Discussion

The results of the present study demonstrate that NAFLD animal models (i.e., HFD and ob/ob mice) did not present altered expression patterns, for constitutively expressed proteins. On the other hand, there were alterations in the constitutive gene expression of several reference genes when the results from the HFD and ob/ob groups were compared. However, there was a considerable increase in *α*-actinin expression levels in the livers of the ob/ob mice when compared to SD controls.

Reference proteins and genes are selected based on the premise that these normalization markers are expressed in all tissues and that the expression of these molecules does not vary under experimental or pathophysiological conditions. However, there are examples of experimental conditions, cell types, and development stages, as well as other conditions such as with some tumors, schizophrenia, and liver disease that have been shown to promote changes in the expression levels of these genes and proteins [2–4, 14]. Therefore, researchers must choose reference genes carefully, so as to avoid misinterpreting results.

More specifically, there is evidence demonstrating that genes often used for data normalization (i.e., *β*-actin, GAPDH, and 18S rRNA) display variable expression levels in the models of hepatitis C virus infections [15–17]. In addition, Boujedidi et al. [18] showed that the levels of constitutively expressed genes *β*-actin and GAPDH were also quite variable in patients with alcohol-induced liver injury.

An experimental condition that has received little attention, with respect to reference genes, is NAFLD. Bruce et al. [5] analyzed 6 genes and proposed that, among these, GAPDH and B2M were the most stable genes in NAFLD, while CYC1 and EIF4A2 were the least stable. Additionally, in the nonalcoholic steatohepatitis (NASH) model, the YWHAZ and ACTB genes were found to be the most stable and the CYC1 and B2M genes were the least stable. However, it was also found that there was some variability in the constitutive expression of some genes and proteins in the control animals, as well. Taking all of this into consideration, it was ultimately concluded that the most stable constituents were YWHAZ and CYC1, while the least stable constituents were EIF4A2 and ACTB.

The results of the present study, using animal models of NAFLD, were similar to those of Bruce et al. [5]. Both studies were unable to detect any significant differences in the GAPDH and B2M gene expression levels between HFD and SD control mice. However, when performing the same analytical procedures with liver samples from ob/ob mice, it was found that the level of GAPDH gene expression was significantly reduced when compared to HFD mice (Figure 2).

Furthermore, the constitutive expression of *β*-actin, *α*-tubulin, RPL37a, and *α*-actinin genes was not altered in relation to control, but did display significant differences when comparing the two NAFLD models. Thus, these reference genes, with the exception of *α*-actinin (*p* < 0.053), should be considered to be good and reliable data normalizers, when comparing each individual model (HFD or ob/ob) with an adequate control. On the other hand, when comparing the two NAFLD models with respect to the control, B2M should be considered the best choice.

Similar to reference genes, which are used to as controls for gene expression analyses, reference proteins, also called housekeeping proteins, are also used as internal controls and serving to normalize the SDS-PAGE or immunoblot data. Examples of such proteins include *β*-actin, GAPDH, *β*-tubulin, and others such as COX-IV and cyclophilin [19], which have all been shown to be expressed at substantial and unaltered levels. As shown in Figures 3(a) and 3(b), Ponceau S, which has been used for qualitative assessments of transfer efficiency, as well as gel loading consistency [20], allows for a quick and efficient evaluation of sample protein content.

Previous studies have shown that the protein expression levels of *β*-actin, GAPDH, and *β*2-microglobulin changed under some experimental conditions, as well as in cases of normal versus inflamed gut. In addition, GAPDH levels are altered in some defective and transfected cell lines and have also been reported in patients with melanoma [2, 21].

In this study, both the Ponceau S staining intensity and immunoblot results demonstrated that there were no significant differences in *β*-actin, *α*-tubulin, or *α*-actinin protein expression levels between or among the groups (Figure 2). In addition, it was found that GAPDH levels, of the control and HFD groups, were slightly altered (*p* < 0.06), but failed to reach a level of significance (Figure 2).

## 7. Conclusions

Based on our results, it is concluded that there are some alterations in the expression levels of reference genes and proteins in the two NAFLD animal models utilized in this study. It is highly recommended that selection of the reference gene(s) be performed carefully when directly comparing these two models. On the other hand, when only comparing one of the models with a suitable control, there are a variety of options available. Our study showed that all constitutive proteins analyzed are good parameters for studies comparing the two animal models (HFD and ob/ob); however, in relation to gene expression, the constitutive gene most suitable was B2M; since there was no difference between the models, we advise you to use it for your analysis.

Finally, it is recommended that the results of this study be taken into consideration when working with these models, so as to ensure that the obtained results are reliable and can be interpreted correctly.

## Data Availability

The data used to support the findings of this study are included within the article.
